# Can low-carbon cities pilot policy promote enterprise sustainable development? Quasi-experimental evidence from China

**DOI:** 10.1371/journal.pone.0301317

**Published:** 2024-05-02

**Authors:** Bowen Zheng, Xiaoyu Wu, Xiaotong Huo, Shuyang Wang

**Affiliations:** School of Business, Macau University of Science and Technology, Macau, China; Sichuan Agricultural University, CHINA

## Abstract

With the predicament of sustainable improvement in traditional cities, the low-carbon city pilot policy (LCCPP), as a novel development mode, provides thinking for resolving the tensions of green development, resource conservation and environmental protection among firms. Using Chinese A-share listed companies panel data during 2007–2019, this study adopts the difference-in-differences model to explore the impact of LCCPP on firm green innovation. Based on theoretical analysis, LCCPP-driven environmental rules have the impact of encouraging business green innovation. The relationship between LCCPP and green innovation is strengthened by external media attention and organizational redundancy resources. The mechanism study shows that the incentive effect of LCCPP on firm green innovation is mainly due to the improvement of enterprises’ green total factor productivity and financial stability. In addition, the heterogeneity analysis shows that the LCCPP has significantly positive effects in promoting green innovation in high-carbon industries and state-owned enterprises. This research contributes to the understanding of city-level low-carbon policies as a driving force for corporate green innovation, offering practical implications for policymakers and businesses striving for sustainability.

## 1 Introduction

An increasing number of organizations have emphasized green innovation during the last few decades to ensure sustainable development [1–3]. For example, many companies focus on recycling and alternative sustainable energy [4,5]. From a micro perspective, green innovation has become an essential driver of green development, helping to fight the battle against pollution and promoting Outstanding economic growth. In contrast, at the macro level, green innovation is essential for enterprises to improve their competitive advantage, accelerating energy conservation and emission reduction, pollution prevention, resource recycling, and promoting green transformation [6]. Green innovation improves the quality of green products, which helps develop better commodities [7], and improves environmental growth while enhancing financial performance and sustainable development [8,9].

Scholars have studied the contributing factors and effects of GI. Environmental regulation, market demand, political capital, and intra-firm measures are some of the factors that have been the subject of studies. [10–12]. Others have explored the effects of green innovation, including financial performance, social performance, and environmental performance [13–15]. However, a few have focused on the drivers of corporate green innovation from the government’s low-carbon development policy perspective, and they have shown that green innovation relies on policy support and incentives from microeconomic agents themselves [16]. Furthermore, few studies have investigated the contextual conditions of the relationship between LCCPP and corporate green innovation. The study fills in the gaps in the literature that keep us from comprehending how green innovation benefits businesses.

To fill these gaps, this study has the following analyses: First, as a tool for environmental regulation, low-carbon city policy has created a strong foundation for low-carbon urban growth [17]. The implementation effects of the pilot low-carbon city policy are examined from the perspective of corporate green innovation, which provides a quantitative scientific basis for evaluating this comprehensive environmental policy and is a valuable addition to the relevant literature on the implementation effects of this policy [18]. Second, this study extends the above relationship boundaries by examining the moderating effects of external media attention and redundant resources within the organization on the benchmarking relationship. Third, From the two perspectives of boosting green total factor productivity and business financial stability, we explore the impact mechanisms of LCCPP procedures promoting green technological innovation. Fourth, we further distinguish between industry carbon intensity and enterprise ownership to identify the direction of the pilot policy in promoting green technological innovation and the industries and enterprise subjects that play a role. Fifth, we collected Chinese listed companies’ patent data, identified different types of green patent application data through patent classification numbers, and organized and constructed a unique green patent database to reflect China’s low-carbon green transformation and technological progress from the micro-level. Sixth, we further distinguish between industry carbon intensity and enterprise ownership to identify the direction of the pilot policy in promoting green technological innovation and the industries and enterprise subjects that play a role.

Moreover, government policies for low-carbon development as a driver of corporate green innovation have received limited attention in prior research, and there has been little study of the contextual conditions in this relationship. Therefore, this paper explores the impact of China’s LCCPP on corporate green innovation and its related mechanisms, framing the discussion within the context of global efforts towards environmental sustainability. The overarching research question investigates whether these policies effectively promote sustainable practices in businesses. To answer this, we adopt a multi-faceted approach, analyzing various aspects such as green total factor productivity and financial stability in enterprises. This study methodology employs a difference-in-differences model, analyzing data from Chinese A-share listed companies from 2007 to 2019.

This study has the following contributions. First, we examine the impact of LCCPP on corporate green innovation and find that LCCPP promotes green technology innovation. The literature emphasizes the significance of strengthening patent protection while also enhancing the external institutional environment [19,20]. For example, the research discusses that the external institutional environment plays an important role in increasing the pressure for carbon emission reduction, and prompting firms to implement green innovation.

Second, this study compensates for the effect of carbon emission reduction on stimulating green innovation in enterprises. Past research has mainly studied the impact of green innovation on low-carbon development [21]. This study provides a new perspective for exploring the effects of low-carbon development policy on corporate green innovation which in line with the effect of innovation compensation brought up by traditional study [22]. This paper shows an essential interpretation of the role of companies that endure the effects of environmental regulations focused on low-carbon development (i.e., external media attention and internal organizational redundancy resources). It can be seen as a further development of Porter’s hypothesis for the era of sustainable development, adding to the increasing amount of knowledge about how carbon reduction policies affect business operations [23,24] and advancing our understanding of how companies can successfully innovate business strategies to comply with environmental regulations. Additionally, this study covers a research gap on how contextual factors affect the relationship between LCCPP and corporate green innovation by incorporating external media attention and internal organizational redundancy resources into the research framework. This study proposes that the LCCPP promotes the micro-mechanisms of firms’ GI growth, revealing green total factor productivity and financial stability as the two critical paths that advance the effectiveness of the policy.

The study is organized as follows: after this introduction, we delve into the literature review and hypothesis development, followed by a detailed description of the research design. The empirical testing and results are presented, culminating in a discussion and implications that synthesizes our findings and outlines their implications for policy and practice.

## 2 Literature review and hypotheses development

### 2.1 Literature review

Despite previous research on the effect of environmental policy on green innovation in enterprises and the implementation of LCCPP, our understanding of the specific techniques by which pilot low-carbon policies affect enterprise-level green innovation is still limited. Businesses only choose the best investments in green innovation based on static resource restrictions. Environmental restrictions are thought to increase the financial burden on businesses, affecting how they allocate resources and decreasing their potential for innovation [25]. However, from a dynamic standpoint, modest environmental regulation can have an impact on regulated enterprises in the form of "innovation compensation". Numerous studies [26,27] have demonstrated that enterprises may entirely counterbalance the negative effects of the financial burden of environmental regulation by improving their capacity for green innovation, which is in line with Porter’s thesis [28].

Promoting green innovation is the objective of the low-carbon development policy The majority of studies on green innovation concentrate on variables that affect how green innovation turns out, including environmental laws [29], economic agglomeration [30], economic growth [31], carbon pricing procedures [32], and energy shortages [33]. Due to its influence on green innovation, LCCPP has become an increasingly important environmental regulatory policy in recent years. However, the existing literature pays less attention to the relationship between LCCPP and green innovation.

Prior research has mixed evidence on the impact of low-carbon development policy on green innovation. On the one hand, environmental measures for low-carbon growth have a compensatory impact in promoting green innovation and resolving the capital-labor mismatch, particularly for highly productive enterprises [34]. On the other hand, during the implementation of the low-carbon development policy, construction projects in China have been "weakly decoupled", and carbon emissions are no longer detached from dependence on electricity consumption at some stage of new urbanization [35]. As a result, the development of low-carbon projects has not been successful in lowering carbon emissions and fostering economic growth [36].

The literature has also looked at how low carbon development policy affects the economy. Early research and later studies concentrated on calculating the economic costs of carbon emission reductions and eliminating economic expenses, however, more recent studies have concentrated more on the effects of carbon reduction policy on business operations. They believe that to satisfy the demands of reducing carbon emissions and improving the supply chain’s green development, businesses and the supply chains they operate within should support policies like green innovation. Based on the above literature, this study tries to analyze the influence of LCCPP as an essential regulation policy on enterprise green innovation and its mechanism. Specifically, this section reviews existing literature on low-carbon policies, green innovation, and sustainable development in enterprises. While there is extensive research on environmental policies and corporate sustainability, a notable gap exists in understanding the specific impact of city-level low-carbon policies on enterprise innovation and productivity. This study aims to fill this gap by analyzing the effects of China’s LCCPP on green innovation in enterprises, particularly focusing on the enhancement of green total factor productivity and financial stability. We develop hypotheses to test these relationships, drawing on theoretical frameworks from environmental economics and corporate sustainability literature. This approach will provide new insights into the effectiveness of local environmental policies in driving sustainable business practices.

### 2.2 Research hypotheses

#### 2.2.1 Low carbon city policy and enterprise green innovation

The Low Carbon City Pilot Policy (LCCPP) is a city-level environmental regulation policy proposed to implement China’s climate action goals. The LCCPP aims to improve energy efficiency, energy saving, and emission reduction in the production process, as well as the transformation and upgrading of industries to a low-carbon direction, thus reducing the carbon intensity and total carbon emissions of the city as a whole.

This process is accompanied by technological innovation in enterprises, which leads to the improved existing technologies and the development of green technologies that meet the needs of low-carbon development, i.e., the realization of the "Porter hypothesis". The Porter hypothesis suggests that environmental regulation affects firms’ production decisions in two ways. First, Environmental legislation will increase the cost of emission reduction and pollution management, which may cause businesses to reduce their short-term R&D expenditure or switch to other types of investment. Prior research shows that firms have to make final decisions on investments when facing environmental policy [37], including increasing technology-based investments to upgrade existing technologies and increasing financial-based investments to diversify their operations. Second, well-designed environmental policies generate innovation compensation effects that enhance firms’ productivity and economic gain. Firms are motivated by regulatory pressure to innovate to increase energy efficiency and reduce emissions from their manufacturing processes to achieve the necessary regulation emission standards, which have positive effects on the environment and the economy. As government-implemented environmental regulation measures, therefore, LCCPP may affect business technological innovation, normally relying on the balance between the “compliance cost” influence and the “innovation compensation” [38].

Without the LCCPP system, businesses may concentrate on maximizing revenues or use end-of-pipe solutions while increasing manufacturing scale to cover the cost of regulations. Enterprises are more willing to gain sustainable competitive advantages through innovation as a result of stronger environmental legislation, stricter enforcement, and rising regulatory costs, and green innovation as a corporate social responsibility aids in developing positive relationships with stakeholders and gaining access to outside resources [39,40]. The “innovation compensation” effect is greater than the “compliance cost” effect, which shows that LCCPP encourages green innovation. These studies show that more effective environmental regulation encourages businesses to adopt green innovation [41], and the following hypothesis is proposed.:

Hypothesis l: The low-carbon city pilot policy has a significant impact on enterprise green innovation.

#### 2.2.2 Moderation effect of media attention

Based on contingency theory, the external environment is the most crucial variable affecting a firm’s production and operation process [42]. Prior research also confirms that the external environment significantly influences a firm’s strategic decision-making on the development of digitalization. External media attention is an essential medium for market information transmission in the external environment. For example, it supports investors in improving their awareness and provides more comprehensive information for investment decisions. Media reports on corporate information also influence investors’ decision-making behavior [43]. As a result, media attention is crucial for both investors and managers [44], The media has the dual function of information dissemination and corporate governance [45], and is an essential channel for stakeholders to understand the enterprise. The moderating effect of media attention on the relationship between LCCPP and green innovation is reflected in the followings: First, according to prior study [46], media coverage alleviates information asymmetry, lowers the cost of finding information, and influences businesses’ use of green innovation. It also effectively communicates information to stakeholders. The effect of media attention is primarily influenced by stakeholders’ expectations and perceptions [47]. Indeed, the media is a tool to guide public suggestions. It helps increase the exposure of green innovation information, enables enterprises to gain external legitimacy, and builds a superior competitive position in the market. Relying on the information delivered by enterprises’ innovation on media, it is more helpful for LCCP to promote firms’ implementation of green innovation strategies.

The media performs a critical monitoring function in external corporate governance. Great media attention means that it generates high public opinion pressure. To increase public knowledge of environmental protection and encourage participation in environmental control by challenging pressure from high-carbon products and public opinion, low-carbon city policies force businesses to publish specified information. In turn, it plays a supervisory and restraining role in the behavior of firm management, prompting them to make strategic decisions on considering the low-carbon city policy. The "regulatory effect" of the policy is used to promote corporate strategies and enhance the transition of cleaner production and green manufacturing. In addition, the media unveils the message that green innovation draws widespread attention from society, which can significantly influence the reputation of enterprises and the investment activities of institutional investors and other stakeholders. Therefore, this study argues that the media can monitor corporate information transparency and stimulate stakeholders’ attention to corporate green innovation in the context of LCCPP, strengthening the positive relationship between LCCPP and corporate green innovation. Based on this, the next hypothesis is suggested.

H2: Media attention positively moderated the relationship between low-carbon city policy and corporate green innovation.

#### 2.2.3 Moderation effect of organizational redundant resources

Redundant resources are considered resource buffers within the organization, it’s a necessary resource guarantee for strategic innovation [48]. Studies have shown that firms with more redundant resources have a greater risk-taking capacity and are proactive, preferring high-risk and untried activities to financially constrained firms [49]. At the organizational level, high-quality innovation emphasizes the need for organizations to invest more resources to enhance their innovation operations [50]. Corporate redundancy resources play an essential role in green innovation. According to prior study [51], having more redundant organizational resources enables businesses to take on riskier tasks and helps them look for and take advantage of bigger possibilities and issues. We argue that firms with more redundant resources enhance the value effect of LCCPP on corporate green innovation.

First, abundant redundant resources motivate firms to engage more in remote search (where firms seek information outside their current market and technology domain) [52]. It increases the likelihood of executives being exposed to and learning from green technologies. A strong resource base provides a good foundation for applying green technologies for high-quality innovation and enables companies to cope with the riskiness and uncertainty in the green innovation process. Redundant resources allow enterprises to integrate new technologies and develop new capabilities.

**Fig 1:**
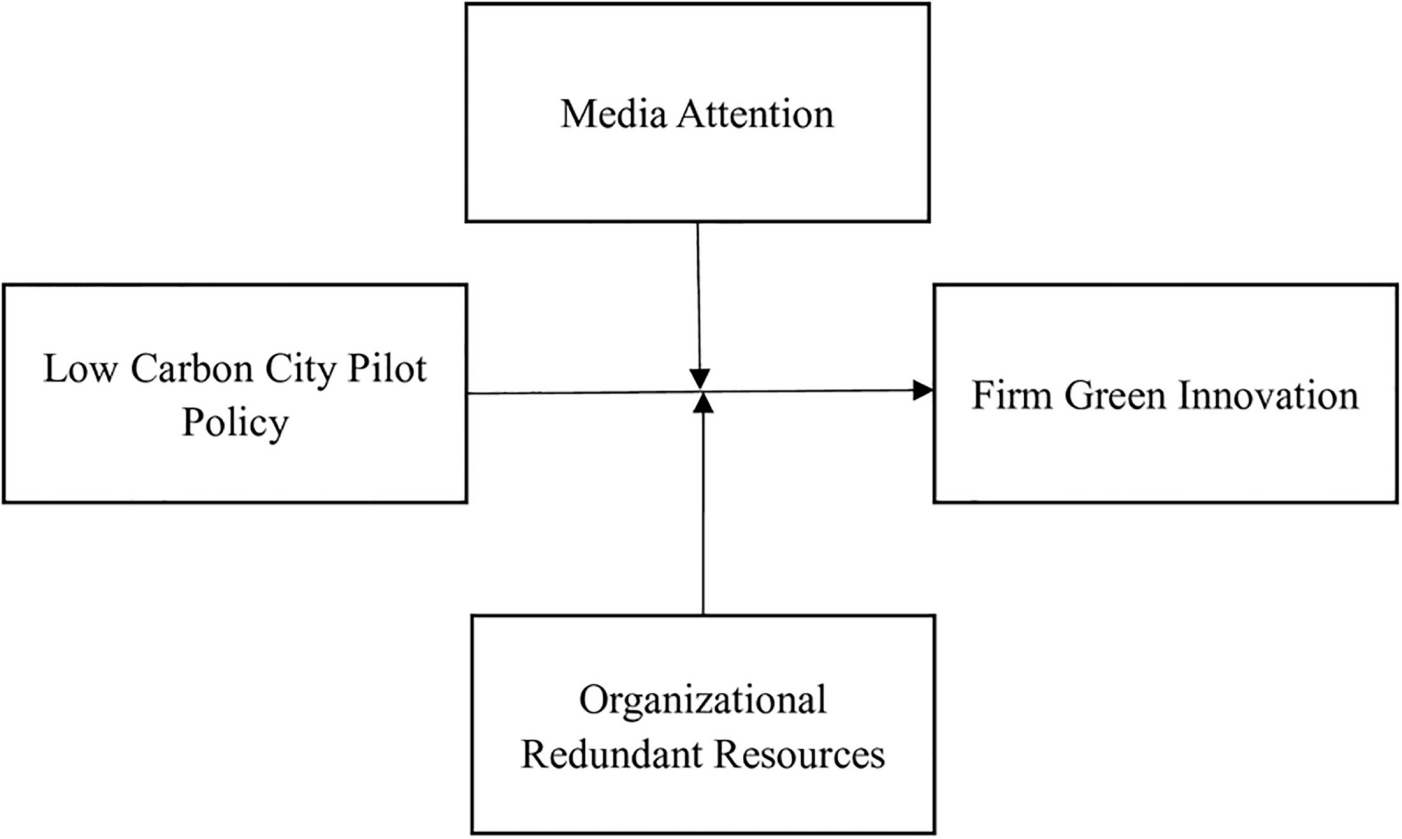
Theoretical research model diagram.

Second, executives enjoy higher flexibility and more choices when resources are abundant. It can empower executives with higher willingness and motivation to operate with resources regarding green innovation. Redundant resources effectively reduce infighting and conflict among executives and mitigate disagreements over resource negotiation and allocation. Green innovation takes time and involves continuous resource investment, the long-term commitment of executives is crucial. Overall, redundant resources lay the foundation for organizations to apply green technologies in the context of low-carbon city development, grant executives higher organizational autonomy, effectively enhance their ability to implement green innovation strategies. Based on this, the following hypothesis is proposed. The research model is shown in Fig 1.

H3: Organization redundant resources positively moderated the relationship between low-carbon city policy and corporate green innovation.

## 3 Research design

### 3.1 Data and samples

The sample period for this study begins in 2007 and lasts through 2019 due to problems with data availability while compiling data at the firm and municipal level in China. the decision to begin in 2007 was made since listed firms in China began using the new accounting rules on January 1, 2007; this achieved convergence with the International Financial Report Standard (IFRS). Moreover, the data selection until 2019 was driven by data availability and consistency, and to assess the long-term effects of earlier low-carbon policies, excluding recent policy changes and global events. This time-frame also avoids the confounding impacts of the COVID-19 pandemic, ensuring a clearer analysis of low-carbon policies on enterprise sustainability without the distortions from these extraordinary external factors.

Among these, patent information data of listed companies were drawn from the State Intellectual Property Office of China (SIPO). Data at the city level were taken from the yearbook of China City Statistical. To locate specific missing numbers, statistical bulletins on each company’s or city’s National Economic and Social Development were sorted. The China Stock Market & Accounting Research databases (CSMAR) provided information on listed businesses. The financial sector’s sample of businesses was left out of this study due to the major distinctions between it and other sectors. All continuous variables were winsorized at this study’s 1st and 99th percentiles to eliminate the influence of outliers.

### 3.2 Measurements

To encourage green innovation and the transformation of businesses, China’s National Development and Reform Commission (NDRC) introduced the nation’s first batch of low-carbon provinces, regions, and cities in 2010. To reduce carbon emissions intensity and aggressively pursue low-carbon green development, the NDRC mandated every pilot zone to propose action goals, essential duties, and specific ways to manage greenhouse gas emissions in the zone. Pilot projects’ second and third waves were introduced in 2012 and 2017. In the first package of experimental zones, the NDRC named five provinces, two local governments, and six prefecture-level cities.

#### 3.2.1 Dependent variables

Referring to the prior literature on green innovation (GRInno) [2], the dependent variable in this study is the number of green patent applications of listed enterprises. The indicators of the number of green patent applications include the total number of green patents (LnTotal), including the number of green invention patents (LnInva) and the number of green utility patents (LnUma). The number of green patents applied by listed enterprises in the current year is logarithmically processed.

#### 3.2.2 Independent variable

For the independent variable of the Low Carbon City Pilot Policy (LCCPP) [17], the core explanatory variables of the difference-in-difference (DID) method are represented by constructing interaction items (Pilot×Post). Pilot denotes a dummy variable for the pilot areas of low-carbon cities, taking the value of 1 if the city or province is a pilot area in the first three batches of policy announcements and 0 otherwise. Post is a dummy variable before and after the policy pilots, taking the value of 1 during the pilot period of low-carbon cities (i.e., after 2012) and 0 during the non-pilot period.

#### 3.2.3 Control variables

Some control variables are chosen to take into account elements at the city and enterprise levels that could have an impact on the degree of a company’s green innovation. First, corporate characteristics and governance structure indicator variables. (1) Size: Larger firms with a greater incentive to enhance environmental governance. firm size is measured by the natural logarithm of total assets. (2) Age: It demonstrates how time influences green image. Firm age is measured by the years the firm has been established. (3) Board: It represents the logarithm of the whole number of board members. (4) Independent directors (Inde): It is calculated as the ratio of independent directors to all other directors. (5) Largest shareholder holding (Top1): The ratio of the largest shareholder’s shareholding to the total number of shares is used to calculate it.

Second, Corporate Financial Indicator variables. (6) Financial leverage (Lev): It reflects the market’s assessment of a firm’s creditworthiness [53], and a moderate level of indebtedness allows firms to have more funds for innovative activities. Total liability divided by total assets measures it. (7) Profitability (ROA): Net profit divided by total assets, or ROA, is a proxy indicator of the firm’s performance.

Third, city-level Indicator variables [17]. (8) foreign investment (FDI): It is the ratio of the total output value of foreign-invested industrial enterprises to the total industrial output value of the region. (9) government intervention (Gov): The share of public revenue in the city GDP at the prefecture level. (10) Investment level of city fixed assets (Tangcitybl): It indicates the share of fixed assets in GDP at the city level.

#### 3.2.4 Moderating variables

Media Attention (Media) is the first moderator, it is represented by the media coverage, including network media coverage and press coverage from CNRDS [45]. Among them, online financial news includes news report data from more than four hundred important online media; the most important are those news reports from twenty mainstream online financial media.

Organizational Redundant Resources (ORS) are another moderator. Following prior research [54], the cash ratio determines the organizational redundant resources. A higher ratio means more organizational redundant resources.

#### 3.2.5 Mediating variables

Green total factor productivity (GTFP). According to the environmental technology function [55], urban green total factor productivity growth is measured under the global reference data envelopment analysis framework, which integrates the super-efficient SBM model considering non-desired outputs and the Malmquist productivity index [56]. The global reference approach addresses the issues of infeasible solutions and incomparability across periods by building the optimal production frontier applying the input-output data from each decision unit in the examination period and measuring all decision units in various periods under the global optimal production frontier. After obtaining the directional distance function by linear programming, this study follows prior research [57] to obtain the green total factor productivity of the ML index from period t to period t+1. The indexing measurement requires solving four directional distance functions under constant and variable scale payoff with the help of the linear programming method, among which two linear programs solve for the current period directional.

Financial Stability (FS). The Altman’s index is measured by the accounting method of Gentry and Shen [58]., which reflects the company’s financial distress. Z is calculated as (1.2 × working capital / total assets) + (1.4 × retained earnings/total assets) + (3.3 × income before interest expense and taxes/total assets) + (0.6 × market value of equity/total liabilities) + (1.0 × sales/total assets). Firms with Z-scores below 1.81 are classified as financially distressed. The judgment criteria are Z>2.67 for good financial stability, and 1.81<Z<2.67 is a grey area, indicating that the financial situation is precarious and the possibility of financial distress is high.

### 3.3 Model design

This study tests whether a LCCPP can promote green innovation. The most effective method in the literature on policy implementation effect assessment is the Difference-in-Differences (DID) model [59]. Thus, to test the judgment of Hypothesis 1, the following benchmark model settings are as follows:

$${\text{GRInno}}_{\text{it}+1}=\alpha_{0}+\alpha_{1}\times {\text{Pilot}}_{\text{r}}\times {\text{Post}}_{\text{t}}+\alpha_{\text{k}}\sum {\text{Control}}_{\text{it}}+\delta_{\text{rt}}+\mu_{\text{jt}}+\gamma_{\text{i}}+\varepsilon_{\text{ijrt}}$$ (1)

To test hypotheses 2 and 3, we construct regression model to test the moderating effects.


$$\begin{array}{cc} {\text{GRinno}}_{\text{I},\text{t}+1} & =\beta_{0}+\beta_{1}\times {\text{Pilot}}_{\text{r}}\times {\text{Post}}_{\text{t}}+\beta_{2}\times {\text{Media}}_{\text{I},\text{t}}+\beta_{3}\times {\text{Pilot}}_{\text{r}}\times {\text{Post}}_{\text{t}}\times {\text{Media}}_{\text{it}}\\ & +\beta_{\text{k}}\sum {\text{Control}}_{\text{i},\text{t}}+\text{Ind}+\text{Year}+\text{Prov}+\varepsilon_{\text{i},\text{t}} \end{array}$$ (2)

$$\begin{array}{cc} {\text{GRinno}}_{\text{I},\text{t}+1} & =\beta_{0}+\beta_{1}\times {\text{Pilot}}_{\text{r}}\times {\text{Post}}_{\text{t}}+\beta_{2}\times {\text{ORS}}_{\text{I},\text{t}}+\beta_{3}\times {\text{Pilot}}_{\text{r}}\times {\text{Post}}_{\text{t}}\times {\text{ORS}}_{\text{it}}\\ & +\beta_{\text{k}}\sum {\text{Control}}_{\text{i},\text{t}}+\text{Ind}+\text{Year}+\text{Prov}+\varepsilon_{\text{i},\text{t}} \end{array}$$ (3)

In the benchmark analysis, where i, t, and r represent the enterprise, time, and city, respectively, and the *α*_*1*_ coefficient of the term *Pilot*_*r*_ × *Post*_*t*_ of this study. This coefficient reflects the impact of the LCCPP on green patent applications after the DID method between before and after it was implemented, as well as across regions that are participating in the pilot and those that are not. If *α*_*1*_ is significantly positive, it means that the LCCPP helps promote green innovation activities of enterprises in the pilot areas, and ∑*Control* denotes the summation of the model’s control variables. In addition, the model controls for the time-varying industry and provincial fixed effects.

### 3.4 Descriptive statistics of variables

The explanatory variables, major explanatory variables, control variables, and moderating variables’ descriptive statistics are shown in Table 1. The greatest value of the proportion of the overall amount of green patents (LnTotal) according to the descriptive statistics is 3.714, the average value is 0.414, the median value is 0, and the minimum value is also 0. This demonstrates that there are many variations in the overall amount of green patents and that many listed companies have not yet submitted their applications. The mean and the standard deviation values of LCCPP are 0.4838 and 0.4997, indicating the implementation of LCCPP across the country. The mean value of media attention is 4.892, and the standard deviation is 1.125. This shows a high level of media attention to listed companies. The mean value of organizational redundant resources is 1.007, and the standard deviation is 1.860. This indicates that the company has a relatively reasonable level of cash ratio.

**Table 1:** Descriptive statistics.

VarName	Obs	Mean	SD	Min	Median	Max
LnTotal	20850	0.419	0.832	0.000	0.000	3.714
LnInva	20850	0.283	0.660	0.000	0.000	3.258
LnUma	20850	0.253	0.603	0.000	0.000	2.890
LCCPP	20850	0.484	0.500	0.000	0.000	1.000
Media	20850	4.892	1.125	1.946	4.934	7.818
ORS	20850	1.007	1.860	0.019	0.400	12.500
Size	20850	21.939	1.285	19.148	21.776	25.884
Age	20850	2.819	0.341	1.792	2.833	3.497
Board	20850	2.259	0.180	1.792	2.303	2.773
Inde	20850	0.373	0.053	0.300	0.333	0.571
Top1	20850	0.369	0.151	0.080	0.356	0.754
Lev	20850	0.426	0.211	0.049	0.418	0.998
Roa	20850	0.039	0.064	-0.388	0.039	0.217
Gov	20850	0.110	0.043	0.037	0.102	0.207
FDI	20850	0.029	0.020	0.000	0.028	0.091
Tangcitybl	20850	0.455	0.315	0.000	0.449	1.000

The correlation coefficient of LCCPP and LnTotal is 0.110 in Table 2, and they have a significant positive correlation at the level of 1%, suggesting that LCCPP promotes corporate innovation. H1 is primarily verified.

**Table 2:** Correlation analysis.

	LnTotal	LnInva	LnUma	LCCPP	Media	ORS	Size	Age	Board	Inde	Top1	Lev	Roa	Gov	FDI	Tangcitybl
LnTotal	1															
LnInva	0.929***	1														
LnUma	0.882***	0.698***	1													
LCCPP	0.111***	0.112***	0.085***	1												
Media	0.202***	0.214***	0.168***	0.238***	1											
ORS	-0.058***	-0.049***	-0.065***	-0.065***	-0.037***	1										
Size	0.244***	0.252***	0.225***	0.129***	0.458***	-0.262***	1									
Age	-0.012*	0.007	-0.021***	0.284***	0.119***	-0.181***	0.158***	1								
Board	0.042***	0.052***	0.037***	-0.136***	0.060***	-0.082***	0.258***	-0.005	1							
Inde	0.016**	0.012*	0.018***	0.092***	0.085***	0.017**	0.020***	-0.012*	-0.493***	1						
Top1	0.030***	0.019***	0.044***	-0.005	0.059***	0.056***	0.169***	-0.181***	-0.002	0.055***	1					
Lev	0.064***	0.061***	0.079***	-0.048***	0.089***	-0.535***	0.426***	0.161***	0.163***	-0.021***	-0.044***	1				
Roa	0.041***	0.043***	0.027***	-0.030***	0.075***	0.190***	0.000	-0.109***	0.01	-0.017***	0.157***	-0.350***	1			
Gov	0.066***	0.079***	0.039***	0.395***	0.148***	0.050***	0.116***	0.072***	-0.055***	0.057***	0.070***	-0.033***	0.019***	1		
FDI	-0.022***	-0.014**	-0.025***	-0.092***	-0.018***	0.055***	-0.027***	-0.116***	0.009	-0.006	0.027***	0.010	0.029***	0.224***	1	
Tangcitybl	-0.084***	-0.081***	-0.066***	-0.285***	0.085***	-0.001	-0.059***	-0.114***	0.092***	-0.057***	-0.028***	0.057***	0.003	-0.373***	0.169***	1

Notes:

***, ** and * indicate significance at the 1%, 5%, and 10% levels, respectively.

## 4 Empirical testing

### 4.1 Base regression results

This section examines the quantitative impact of implementing the LCCPP on the green innovation of listed enterprises. The estimation results are shown in Table 3. Column 1 shows the total number of corporate green patent applications, column 2 shows the number of corporate green invention-based patent applications, and column 3 corresponds to the number of corporate green utility model patent applications. Industry, year, and city fixed effects were all controlled in the regression models.

**Table 3:** Baseline model regression results.

	(1)	(2)	(3)
	LnTotal	LnInva	LnUma
LCCPP	0.0525***	0.0553***	0.0300**
	(2.6382)	(3.4910)	(2.0311)
Size	0.1802***	0.1518***	0.1155***
	(26.5282)	(26.6328)	(22.3048)
Age	-0.1867***	-0.1004***	-0.1412***
	(-9.4544)	(-6.5697)	(-9.5148)
Board	0.0913**	0.0861**	0.0458
	(2.1932)	(2.4942)	(1.4936)
Inde	-0.1061	-0.0506	-0.0183
	(-0.8249)	(-0.4893)	(-0.1931)
Top1	-0.1274***	-0.1421***	-0.0409
	(-3.1311)	(-4.3380)	(-1.3411)
Lev	0.1431***	0.0945***	0.1162***
	(4.0610)	(3.3625)	(4.5108)
Roa	0.8685***	0.6591***	0.4700***
	(10.0920)	(9.4395)	(7.8013)
Gov	1.8279***	1.3589***	1.1231***
	(6.5387)	(6.0559)	(5.3862)
FDI	0.7682**	0.8386***	0.1581
	(2.0770)	(2.7766)	(0.5896)
Tangcitybl	-0.0432	-0.0177	-0.0535*
	(-1.1644)	(-0.5922)	(-1.9410)
_cons	-3.9527***	-3.4658***	-2.4843***
	(-21.1002)	(-22.1510)	(-17.6870)
Year	Yes	Yes	Yes
Industry	Yes	Yes	Yes
Province	Yes	Yes	Yes
N	20850	20850	20850
Adj.R^2^	0.1607	0.1465	0.1337

Notes:

***, ** and * indicate significance at the 1%, 5%, and 10% levels, respectively. Parenthetical figures represent t-statistics.

The regression results for the base model using the static effect estimation approach are shown in Table 3. According to column (1), after controlling for the three fixed effects, the double difference term LCCPP coefficient is significantly positive at the 1% level, indicating that implementing the low-carbon city policy can, to some extent, promote green innovation. The implementation of this policy has led to green patent applications. When the dependent variable distinguishes different types of green innovation patents, the coefficients of LCCPP are also significantly positive at the 1% level in columns (2) and (3). The regression results indicate a robust and positive relationship between LCCPP and corporate green innovation, and Hypothesis 1 is supported. This suggests carbon incentives and monitoring measures are valid LCCPP for firms to enhance green innovation.

### 4.2 Moderation test

Table 4 provides the regression results of the moderating effect of media attention and the organization’s redundant resources. Column(1) the term for first-order regulation of media attention, LCCPP*Media, is introduced to examine if there’s a noteworthy linear regulatory impact. The outcome of the regression shows that the LCCPP coefficient stands at 0.0332, manifesting significance at the 10% level. The interaction term LCCPP×Media carries a coefficient of 0.0530, exhibiting significance at the 1% level, indicating that media attention has a significant linear regulation effect on the relationship between LCCPP and corporate green innovation. Meanwhile, column (4) introduces the first-order regulation term of organization redundant resources, LCCPP×ORS, to test whether organization redundant resources have a significant linear moderating effect. The coefficient of the interaction term LCCPP×ORS is 0.0149. It is significant at the 1% level, indicating that organization redundant resources have a significant linear regulation effect on the relationship between LCCPP and corporate GI. In column (2) and column (3), we distinguish different types of green innovation, and the interaction term between LCCPP and media attention is significantly positive (coef. = 0.0676, p<0.01; coef. = 0.0219, p<0.05). This confirmation substantiates the significant positive regulatory influence of media attention on the positive correlation between low-carbon city policy and corporate innovation. H2 is verified. Similarly, In column (5) and column (6), we distinguish between different types of green innovation, and the interaction term between LCCPP and organization redundant resources are significantly positive (coef. = 0.0097, p<0.05; coef. = 0.0143, p<0.01), H3 is supported.

**Table 4:** Moderating tests.

	(1)	(2)	(3)	(4)	(5)	(6)
	LnTotal	LnInva	LnUma	LnTotal	LnInva	LnUma
LCCPP	0.0332*	0.0317**	0.0215	0.0503**	0.0538***	0.0279*
	(1.6907)	(2.0496)	(1.4751)	(2.5358)	(3.4114)	(1.9017)
Media	0.0831***	0.0745***	0.0499***			
	(12.0245)	(12.9782)	(9.6964)			
LCCPP×Media	0.0530***	0.0676***	0.0219**			
	(3.7884)	(5.6381)	(2.0613)			
ORS				-0.0662*	-0.0452	-0.0463*
				(-1.7502)	(-1.4700)	(-1.8713)
LCCPP×ORS				0.0149***	0.0097**	0.0143***
				(2.8937)	(2.2751)	(4.2585)
Size	0.1473***	0.1225***	0.0957***	0.1801***	0.1518***	0.1153***
	(22.1271)	(22.5290)	(18.9930)	(26.4920)	(26.6080)	(22.2517)
Age	-0.1794***	-0.0933***	-0.1371***	-0.1844***	-0.0989***	-0.1390***
	(-9.0942)	(-6.1127)	(-9.2350)	(-9.3331)	(-6.4641)	(-9.3694)
Board	0.0788*	0.0761**	0.0376	0.0903**	0.0854**	0.0450
	(1.9030)	(2.2276)	(1.2312)	(2.1679)	(2.4732)	(1.4676)
Inde	-0.2060	-0.1390	-0.0788	-0.1028	-0.0484	-0.0151
	(-1.6028)	(-1.3523)	(-0.8290)	(-0.7991)	(-0.4682)	(-0.1597)
Top1	-0.1105***	-0.1258***	-0.0313	-0.1276***	-0.1423***	-0.0407
	(-2.7393)	(-3.8834)	(-1.0333)	(-3.1332)	(-4.3388)	(-1.3349)
Lev	0.1277***	0.0771***	0.1087***	0.1485***	0.0983***	0.1190***
	(3.6374)	(2.7577)	(4.2351)	(4.1384)	(3.4367)	(4.5384)
Roa	0.7502***	0.5568***	0.3973***	0.8671***	0.6581***	0.4700***
	(8.7749)	(8.0515)	(6.6247)	(10.0641)	(9.4207)	(7.7802)
Gov	1.8054***	1.3543***	1.1019***	1.8142***	1.3497***	1.1110***
	(6.4778)	(6.0615)	(5.2961)	(6.4889)	(6.0128)	(5.3300)
FDI	0.7695**	0.8071***	0.1750	0.7709**	0.8403***	0.1611
	(2.0891)	(2.6890)	(0.6540)	(2.0846)	(2.7827)	(0.6010)
Tangcitybl	-0.0333	-0.0057	-0.0492*	-0.0445	-0.0185	-0.0546**
	(-0.8996)	(-0.1899)	(-1.7812)	(-1.1995)	(-0.6212)	(-1.9806)
_cons	-3.0846***	-2.7098***	-1.9516***	-4.0004***	-3.4900***	-2.5192***
	(-16.2345)	(-17.5512)	(-13.7005)	(-20.8253)	(-21.7936)	(-17.5685)
Year	Yes	Yes	Yes	Yes	Yes	Yes
Industry	Yes	Yes	Yes	Yes	Yes	Yes
Province	Yes	Yes	Yes	Yes	Yes	Yes
N	20850	20850	20850	20850	20850	20850
Adj.R^2^	0.1680	0.1570	0.1384	0.1608	0.1466	0.1341

Notes:

***, ** and * indicate significance at the 1%, 5%, and 10% levels, respectively. Parenthetical figures represent t-statistics.

### 4.3 Mechanism test

This study argues that LCCPP helps promote firms’ green innovation through a mediating mechanism that enhances their green total factor productivity and financial stability. Therefore, based on the green total factor productivity and financial stability cost, we test the impact of LCCPP on green innovation. The following empirical model is constructed.


$${\text{GRInno}}_{\text{it}+1}=\alpha_{0}+\alpha_{1}\times {\text{Pilot}}_{\text{r}}\times {\text{Post}}_{\text{t}}+\alpha_{\text{k}}\sum {\text{Control}}_{\text{it}}+\delta_{\text{rt}}+\mu_{\text{jt}}+\gamma_{\text{i}}+\varepsilon_{\text{ijrt}}$$ (4)

$${\text{Mediator}}_{\text{it}+1}=\alpha_{0}+\alpha_{1}\times {\text{Pilot}}_{\text{r}}\times {\text{Post}}_{\text{t}}+\alpha_{\text{k}}\sum {\text{Control}}_{\text{it}}+\delta_{\text{rt}}+\mu_{\text{jt}}+\gamma_{\text{i}}+\varepsilon_{\text{ijrt}}$$ (5)

$${\text{GRInno}}_{\text{it}+1}=\alpha_{0}^{\prime }+\alpha_{1}^{\prime }\times {\text{Pilot}}_{\text{r}}\times {\text{Post}}_{\text{t}}+\alpha_{2}^{\prime }\times {\text{Mediator}}_{\text{r}}+\alpha_{k}^{\prime }\sum {\text{Control}}_{\text{it}}+\delta_{\text{rt}}+\mu_{\text{jt}}+\gamma_{\text{i}}+\varepsilon_{\text{ijrt}}$$ (6)

The results of the mechanical tests for the two mediators are shown in Table 5. After controlling for each fixed effect and control variable, the promotion effect of low-carbon city policy on green innovation in column (1) is consistent with the previous results. The table’s LCCPP coefficient in column (2) is significantly positive at the 1% level, indicating that the LCCPP enhances firms’ green total factor productivity. The LCCPP coefficient in column (3) is significantly positive, but the regression coefficient of LCCPP is lower than that in column (1), indicating that the effect of LCCPP on green innovation holds through the mediating mechanism of green total factor productivity. Columns (4) to (5) show similar results for the mediating effect test of the impact of absorptive capacity. Among them, the coefficient of LCCPP in column (4) is significantly positive at the 5% level, indicating that the LCCPP increases firms’ financial stability. The coefficient of FS in column (5) is significantly positive, and the coefficient of LCCPP decreases compared to column (1), which supports the mediating effect of financial stability. In conclusion, the above results suggest that LCCPP promotes green innovation of firms by promoting, among other things, green total factor productivity and financial stability.

**Table 5:** Mechanism tests.

	(1)	(2)	(3)	(4)	(5)
	LnTotal	GTFP	LnTotal	FS	LnTotal
LCCPP	0.0761***	0.2428***	0.0742***	0.0645**	0.0710***
	(3.5187)	(3.5948)	(3.4363)	(2.4173)	(3.2995)
GTFP			0.0354***		
			(3.7923)		
FS					0.4037***
					(9.6824)
Size	0.1870***	0.5381***	0.1632***	-0.0261***	0.1856***
	(23.5899)	(25.1390)	(16.5656)	(-3.3355)	(23.8946)
Age	-0.2021***	-0.2169***	-0.2034***	0.2884***	-0.0357
	(-9.0839)	(-3.5283)	(-9.1535)	(11.9769)	(-1.2968)
Board	0.0941**	0.3553***	0.0924**	0.1238**	0.0927**
	(2.0368)	(2.6417)	(2.0027)	(2.4195)	(2.0227)
Inde	-0.1417	0.8249**	-0.1380	0.0742	-0.2502*
	(-1.0067)	(2.0108)	(-0.9807)	(0.4763)	(-1.7834)
Top1	-0.1269***	0.3510***	-0.1366***	-0.0465	-0.1761***
	(-2.8169)	(2.6493)	(-3.0189)	(-0.9068)	(-3.9353)
Lev	0.1600***	0.1350	0.1329***	-0.1149**	0.1651***
	(3.8089)	(0.9189)	(3.1255)	(-2.0011)	(3.9499)
Roa	0.9777***	5.7516***	0.8912***	0.5210***	1.0428***
	(9.9543)	(13.8091)	(9.0628)	(3.5994)	(10.7061)
Gov	1.8856***	0.1724	1.8835***	-0.1153	1.8293***
	(6.1103)	(0.1729)	(6.1028)	(-0.2877)	(5.9368)
FDI	0.8076**	3.1858**	0.7778*	1.2472**	0.8131**
	(1.9787)	(2.4626)	(1.9054)	(2.4680)	(2.0016)
Tangcitybl	-0.0301	-0.6959***	-0.0285	-0.2763***	-0.0320
	(-0.7329)	(-5.0170)	(-0.6940)	(-5.0315)	(-0.7829)
_cons	-4.0797***	-3.7894***	-3.8498***	3.2865***	-2.9459***
	(-19.4472)	(-6.4975)	(-17.8487)	(15.0607)	(-14.2918)
Year	Yes	Yes	Yes	Yes	Yes
Industry	Yes	Yes	Yes	Yes	Yes
Province	Yes	Yes	Yes	Yes	Yes
N	17437	17437	17437	17437	17437
Adj.R^2^	0.1642	0.5146	0.1647	0.5262	0.1703

Notes:

***, **, and * indicate significance at the 1%, 5%, and 10% levels, respectively. Parenthetical figures represent t-statistics.

### 4.4 Heterogeneity tests

An essential goal of the LCCPP is to explore and conclude how different corporate and industry carbon intensities can reasonably contribute to low-carbon development. We analyze the heterogeneity of the LCCPP in terms of green innovation from the two aspects mentioned above in Table 6. First, the LCCPP seeks to advance all-encompassing low-carbon urban development. One of the essential focuses is the low-carbonization of industrial structure, i.e., the "carbon reduction" and "carbon reduction" of high-carbon industries, such as energy saving and emission reduction, energy efficiency improvement, and implementation of lower-carbon. Therefore, this policy may be more beneficial for high-carbon industries. Second, the ownership attributes of firms have different impacts on their green innovation. The sample is divided into two sub-samples, state-owned and non-state-owned enterprises, on the baseline model to examine whether the LCCPP produces heterogeneous green innovation effects for different industries and business entities.

**Table 6:** Heterogeneity tests.

	(1)	(2)	(3)	(4)
	SOE	Non-SOE	High carbon intensity of the industry	Low carbon intensity of the industry
	LnTotal	LnTotal	LnTotal	LnTotal
LCCPP	0.1377***	-0.0071	0.1122***	-0.0182
	(4.0466)	(-0.2964)	(4.3205)	(-0.4805)
Size	0.2187***	0.1565***	0.1898***	0.1634***
	(21.8948)	(17.2576)	(19.8567)	(18.1812)
Age	-0.1857***	-0.1993***	-0.2274***	-0.1494***
	(-4.9275)	(-8.6102)	(-7.7202)	(-5.7472)
Board	0.0557	0.1010*	0.1452**	0.0698
	(0.8554)	(1.8052)	(2.2830)	(1.3316)
Inde	-0.1236	-0.1892	0.0508	-0.2147
	(-0.6495)	(-1.1118)	(0.2646)	(-1.2631)
Top1	-0.2973***	0.0029	-0.1540***	-0.0506
	(-4.6017)	(0.0550)	(-2.6429)	(-0.9580)
Lev	-0.0738	0.2875***	0.4828***	-0.2278***
	(-1.2830)	(6.2457)	(9.3027)	(-5.0427)
Roa	0.5255***	1.0249***	1.3848***	0.2578**
	(3.5020)	(9.9526)	(11.7625)	(2.2048)
Gov	-0.9468**	3.2023***	2.1664***	0.7175*
	(-2.2425)	(8.8709)	(5.7416)	(1.8641)
FDI	1.5776***	0.2796	0.9235*	0.5356
	(2.6992)	(0.5893)	(1.6603)	(1.0920)
Tangcitybl	0.0621	-0.0722	-0.0298	-0.0809*
	(1.0614)	(-1.5121)	(-0.5669)	(-1.6457)
_cons	-4.1802***	-3.5752***	-4.1338***	-3.1904***
	(-15.1819)	(-13.3392)	(-15.0536)	(-13.4926)
Year	Yes	Yes	Yes	Yes
Industry	Yes	Yes	Yes	Yes
Province	Yes	Yes	Yes	Yes
N	8759	12446	11566	9653
Adj.R^2^	0.2222	0.1491	0.1551	0.1634

Notes:

***, ** and * indicate significance at the 1%, 5%, and 10% levels, respectively. Parenthetical figures represent t-statistics.

It is found that the coefficient of LCCPP on enterprises’ green innovation is positive and significant in the SOE sample in column (1), while in the non-SOE sample in column (2), the coefficient of LCCPP is -0.0071 and is not significant, demonstrating that the SOE sample has a more marked promotion impact of LCCPP on green innovation. There is heterogeneity in the green innovation effect of the LCCPP on enterprises at the level of enterprise ownership attributes. It demonstrates that SOEs focus more on the LCCPP in promoting green innovation in SOEs, while it is not for non-SOEs. It can be explained for several reasons. First, SOEs have stronger path-dependency effects and are generally subjected to stronger environmental regulatory constraints due to their social responsibilities for local economic development [60]. Second, SOEs can use their political capital to obtain more resources for green technology innovation [61]. In addition, SOEs are aware of green innovation more since it contributes to stronger public value attributes.

Columns (3) and (4) show differences in carbon emission industry characteristics. In the sample with high industry carbon intensity, the LCCPP improves the quality of enterprises’ green innovation (coef. = 0.1122, p < 0.01). In contrast, the regression coefficient of LCCPP in the sample with low industry carbon intensity is non-significant (the t-value is only -0.4805), which indicates that the LCCPP is more helpful in promoting green innovation among firms in high-carbon industries. This suggests significant industry heterogeneity in the inducement of green innovation by LCCPP. The effect of green innovation is more obvious in high-carbon industries than in low-carbon industries. This result is consistent with previous studies [62], proving that the major goals of policy regulation and the main providers of green technology innovation are the industries with higher emission intensity.

### 4.5 Robustness test

There are a few presumptions that must be addressed to use the DID approach. Other variables might also have an impact on green innovation. Therefore, we further tested the stability of the research results.

#### 4.5.1 Common trend test

The development patterns or temporal effects between the treatment and control groups are consistent if the treatment group is not susceptible to policy intervention. This test adds the time trend variable period to formula (1) to assess all the years before the policy intervention while controlling the effects of time. The findings of the common trend test are presented in Table 7. Regardless of the control variable, the LCCPP has a considerable beneficial impact on green innovation. The common trend test cannot be explained by the year impact occurring before the policy action, however. The common trend test used in this study on matched cities reveals the consistency over time between the trends of the control group and treatment groups.

**Table 7:** Common trend test.

	(1)	(2)	(3)
	LnTotal	LnInva	LnUma
LCCPP07	0.0341	0.0523	0.0011
	(0.7498)	(1.4731)	(0.0313)
LCCPP08	0.0221	0.0175	0.0238
	(0.5259)	(0.5560)	(0.7582)
LCCPP09	0.0079	-0.0049	0.0265
	(0.1810)	(-0.1533)	(0.7817)
LCCPP10	0.0217	0.0173	0.0203
	(0.5323)	(0.5496)	(0.6680)
Period	0.0229***	0.0120***	0.0149***
	(5.0982)	(3.4547)	(4.2693)
Size	0.1799***	0.1515***	0.1153***
	(26.5078)	(26.5938)	(22.2845)
Age	-0.1863***	-0.1004***	-0.1411***
	(-9.4288)	(-6.5645)	(-9.5078)
Board	0.0903**	0.0847**	0.0450
	(2.1695)	(2.4539)	(1.4688)
Inde	-0.1046	-0.0490	-0.0180
	(-0.8129)	(-0.4734)	(-0.1902)
Top1	-0.1259***	-0.1394***	-0.0401
	(-3.1030)	(-4.2674)	(-1.3186)
Lev	0.1594***	0.0930***	0.1274***
	(5.1262)	(3.7401)	(5.6387)
Roa	0.8746***	0.6646***	0.4737***
	(10.1569)	(9.5107)	(7.8534)
Gov	1.9261***	1.4523***	1.1984***
	(6.9442)	(6.5064)	(5.8037)
FDI	0.7530**	0.8508***	0.1138
	(2.0054)	(2.7652)	(0.4194)
Tangcitybl	-0.0597	-0.0373	-0.0603**
	(-1.5935)	(-1.2358)	(-2.1769)
_cons	-3.9807***	-3.4692***	-2.5031***
	(-21.3547)	(-22.2873)	(-17.9013)
Year	Yes	Yes	Yes
Industry	Yes	Yes	Yes
Province	Yes	Yes	Yes
N	20850	20850	20850
Adj.R^2^	0.1603	0.1460	0.1335

Notes:

***, **, and * indicate significance at the 1%, 5%, and 10% levels, respectively. Parenthetical figures represent t-statistics.

#### 4.5.2 Placebo test

The placebo test is applied for robustness checks by artificially changing the policy implementation’s time point and span. This study randomizes the time points of enactment of this policy (e.g., 2008 year). If the coefficient of LCCPP is insignificant, then low-carbon pilots promote green innovation. If the coefficient is significant, other factors besides low-carbon pilots affect green innovation.

**Fig 2:**
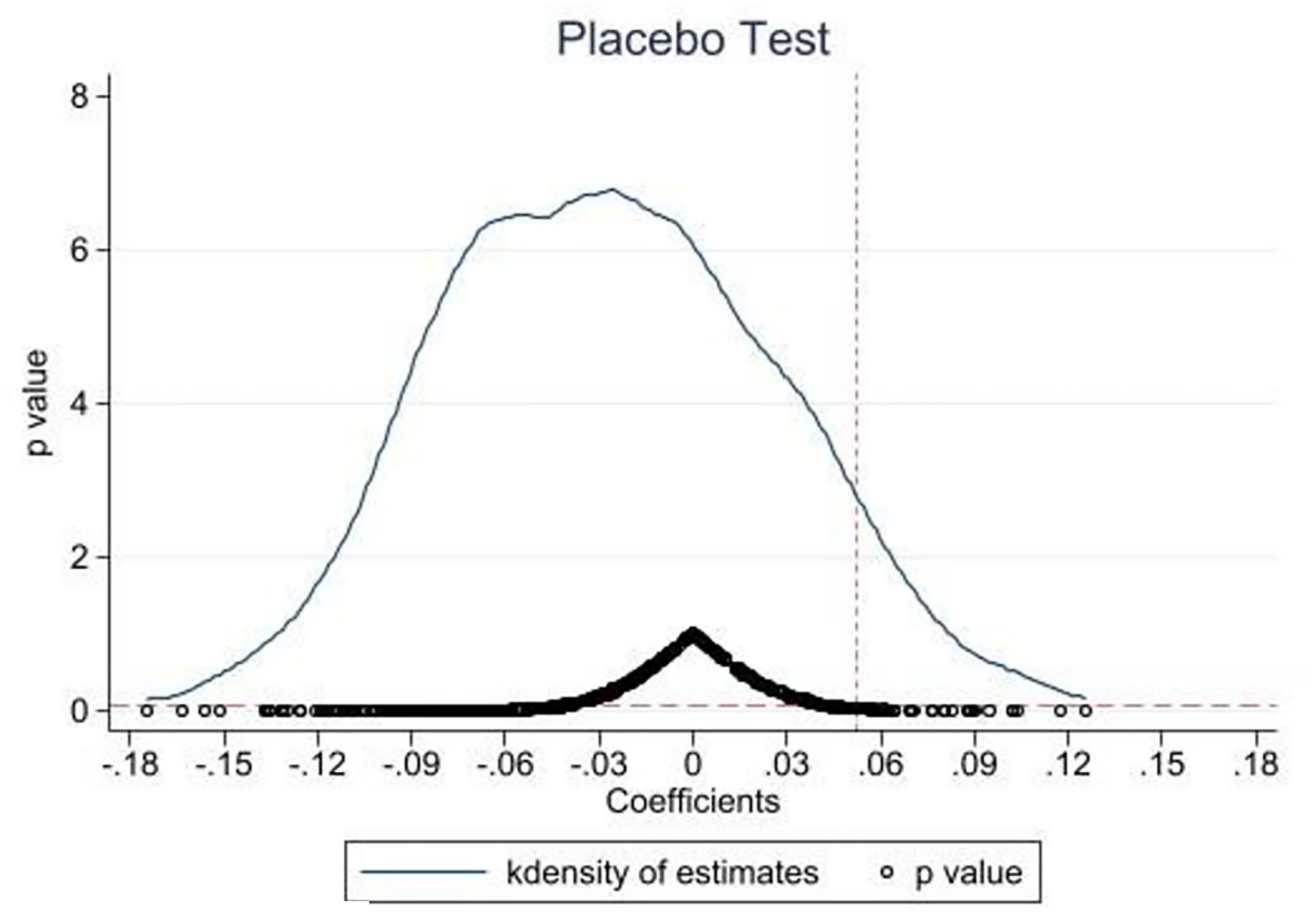
Placebo test.

Fig 2 shows that regardless of changing the time point and span of policy implementation, the coefficient of LCCPP is insignificant, so randomly changing the policy time point has almost no effect on the estimation results, indicating that low-carbon cities indeed bring about green innovation.

#### 4.5.3 PSM-DID

**Fig 3:**
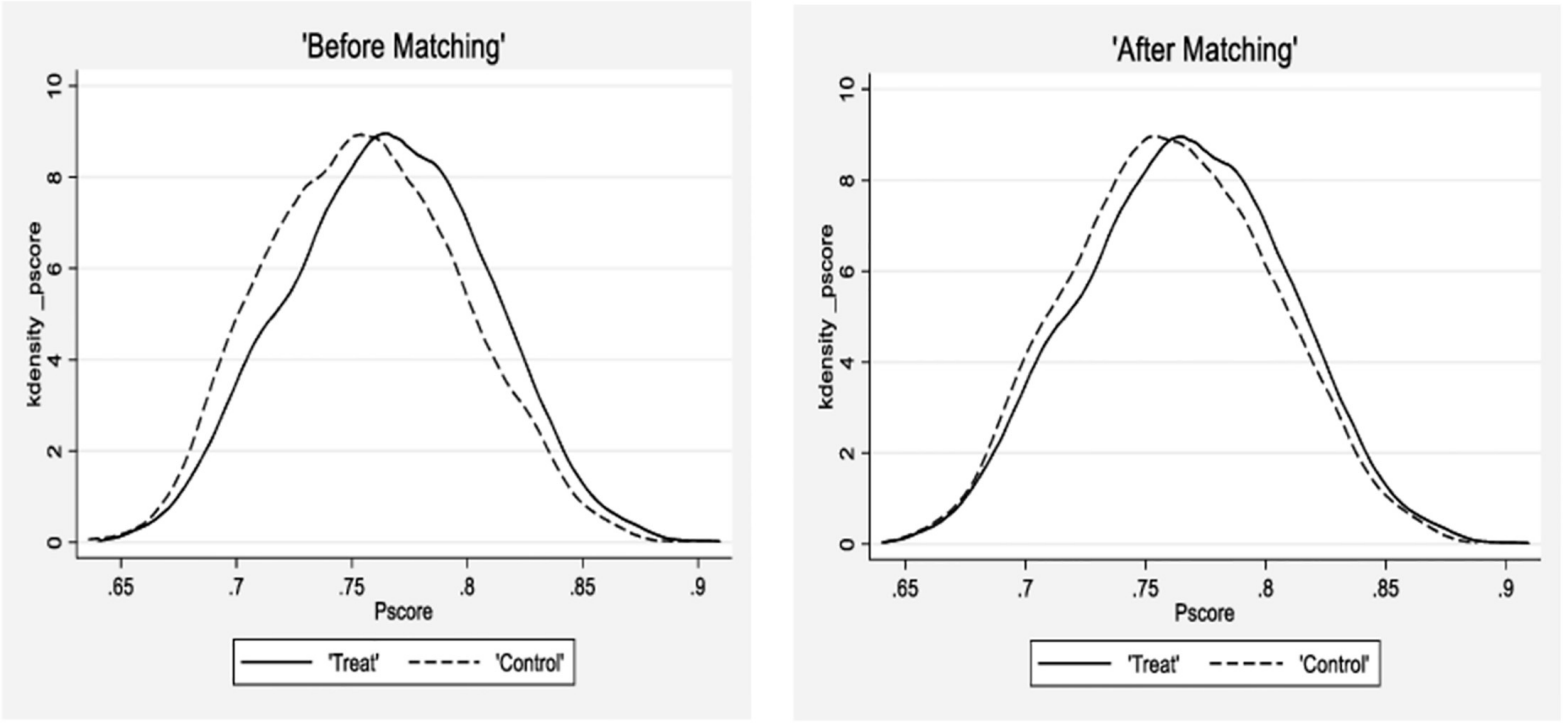
Nuclear density curve before and after matching.

We introduce the PSM-DID method to test the robustness of the basic regression. The treatment group is matched year by year through PSM, according to the 1:1 nearest neighbor matching with put-back sampling. This study’s control variables are selected as matching variables. Then a control group company is matched for each experimental group company according to the matching principle of 1:1. By observing the kernel density matching plots in Fig 3, the kernel density curves of the matched experimental and control groups are relatively close, indicating that the matching effect is good. The results of the PSM-DID regression are provided in Table 8. The findings reveal that the coefficients of LCCPP are significantly positive at the 1% level. This demonstrates that the LCCPP can effectively enhance a firm’s green innovation. This outcome not only supports our primary hypothesis but also corroborates the robustness of the basic regression results.

**Table 8:** PSM-DID tests.

	(1)	(2)	(3)
	LnTotal	LnInva	LnUma
LCCPP	0.093***	0.080***	0.066***
	(3.578)	(3.804)	(3.427)
Size	0.180***	0.161***	0.111***
	(19.949)	(20.906)	(16.280)
Age	-0.215***	-0.125***	-0.164***
	(-7.247)	(-5.327)	(-7.377)
Board	0.219***	0.170***	0.139***
	(3.487)	(3.213)	(3.034)
Inde	0.146	0.144	0.167
	(0.765)	(0.931)	(1.171)
Top1	-0.100*	-0.162***	0.007
	(-1.753)	(-3.493)	(0.159)
Lev	0.325***	0.205***	0.246***
	(7.161)	(5.564)	(7.502)
Roa	1.198***	0.940***	0.653***
	(10.261)	(9.814)	(8.063)
Gov	1.843***	1.469***	0.971***
	(5.141)	(5.055)	(3.648)
FDI	0.474	0.659	-0.039
	(0.859)	(1.425)	(-0.097)
Tangcitybl	-0.028	-0.012	-0.040
	(-0.560)	(-0.303)	(-1.090)
_cons	-4.229***	-3.861***	-2.571***
	(-15.242)	(-16.550)	(-12.406)
Year	Yes	Yes	Yes
Industry	Yes	Yes	Yes
Province	Yes	Yes	Yes
N	12231	12231	12231
Adj.R^2^	0.160	0.148	0.137

Notes:

***, ** and * indicate significance at the 1%, 5%, and 10% levels, respectively. Parenthetical figures represent t-statistics.

#### 4.5.4 The change in the measurement method of corporate green innovation

This study uses the ratio of the number of green patent applications filed by listed companies (RataoTotal), i.e., the ratio of green patents filed by the company to all patents filed by it in the year, and the ratio of two subtypes of green patents -the ratio of the number of green invention-based patent applications (RataoInva) and the ratio of the number of green utility model patent applications (RataoUma), to ensure the robustness of the benchmark analysis. The regression results are shown in Table 9, from which it can be seen that LCCPP still significantly promotes enterprises’ green innovation, indicating that this study’s results are robust.

**Table 9:** Robustness tests.

	(1)	(2)	(3)
	RataoTotal	RataoInva	RataoUma
LCCPP	0.0116***	0.0094***	0.0015
	(2.7924)	(3.0969)	(0.7538)
Size	0.0059***	0.0053***	0.0004
	(5.5352)	(7.1610)	(0.8494)
Age	-0.0429***	-0.0163***	-0.0232***
	(-10.4447)	(-6.0117)	(-10.8886)
Board	-0.0083	-0.0014	-0.0052
	(-1.0940)	(-0.2598)	(-1.3960)
Inde	-0.0971***	-0.0529***	-0.0388***
	(-3.8920)	(-3.0086)	(-3.2860)
Top1	-0.0290***	-0.0231***	-0.0036
	(-3.7011)	(-4.2019)	(-0.9443)
Lev	0.0273***	0.0148***	0.0104***
	(3.4412)	(2.6599)	(2.7535)
Roa	0.0496**	0.0399***	0.0080
	(2.5733)	(2.8167)	(0.8933)
Gov	0.2092***	0.1334***	0.0729**
	(3.3685)	(3.1591)	(2.3678)
FDI	0.0399	0.0592	-0.0134
	(0.5030)	(1.0427)	(-0.3626)
Tangcitybl	-0.0042	0.0003	-0.0033
	(-0.5112)	(0.0450)	(-0.8165)
_cons	0.0221	-0.0533**	0.0617***
	(0.6846)	(-2.3082)	(4.1127)
Year	Yes	Yes	Yes
Industry	Yes	Yes	Yes
Province	Yes	Yes	Yes
N	20102	20102	20102
Adj.R^2^	0.0589	0.0443	0.0549

Notes:

***, ** and * indicate significance at the 1%, 5%, and 10% levels, respectively. Parenthetical figures represent t-statistics.

### 4.6 Discussion of empirical findings

This study finds that the LCCPP significantly enhances firms’ green innovation, echoing [63] findings that policy interventions can stimulate firms’ environmental innovation practices. This study builds on prior research [64] to demonstrate the specific impact of the LCCPP on green innovation in high-carbon industries. The LCCPP promotes more efficient use of resources and reduces environmental impacts, supporting [65] argument for policy-driven environmental benefits. This study also finds that the LCCPP improves financial stability and green total factor productivity, which is consistent with [66] findings on the economic advantages of environmental policies. Overall, LCCPP effectively enhances the environmental and economic benefits of firms.

Moreover, in terms of firm ownership heterogeneity, the LCCPP has different impacts on different firm types. As SOEs have easier access to resources and policy support, LCCPP significantly increases the level of GI of SOEs. This is consistent with prior findings [67] on the policy responsiveness of SOEs. In terms of industry heterogeneity, for example, in high-carbon industries, the policy significantly promotes the development of GI in firms, addressing industry-specific challenges and aligning with green transition goals. On the contrary, in the low-carbon industry sector, the impact of the policy was not significant, suggesting that the policy’s effects are heterogeneous across industries.

## 5 Conclusions, implications, and limitations

### 5.1 Conclusions

Green innovation acts as a crucial catalyst in advancing the low-carbon progression of cities. Utilizing the sample data from Chinese A-share listed firms spanning 2007 to 2019, along with the count of applied green patents by these companies, this research scrutinizes the impact of LCCPP on the company’s green tech innovation. First, the findings indicate that the LCCPP considerably bolsters activities related to green innovation. The parallel trend hypothesis, placebo test, and other measures of green innovation support the findings in the robustness tests. Second, the positive effect of LCCPP on firms’ green innovation is positively moderated by media attention and organizational redundancy resources. The mechanical analysis showed a positive effect of LCCPP on firms’ green innovation, which enhances firms’ green total factor productivity and financial stability. Moreover, the result of the pilot policy promoting green technology innovation is more significant in high-carbon industries and state-owned enterprises. This provides evidence to support that LCCPP exerts governance effects on corporate green innovation, thus further complementing the research on the influencing factors of corporate green innovation.

### 5.2 Theoretical implications

This study has three theoretical implications. First, the results of this study are consistent with Porter’s hypotheses. According to prior research [68], different environmental restrictions and innovations are associated in different ways. Endogeneity is a common problem in hybrid discovery. This can be caused by the endogeneity that comes from measurement error. Prior research has primarily used surveys to quantify reported environmental stringency [69] or the costs of pollution remediation [70]. This may lead to endogeneity issues and systematic measurement mistakes. The failure to include pertinent factors is another source of endogeneity. Our work investigates a perfect environment that enables us to use a thorough and analytical strategy to reduce endogeneity problems. This research establishes a causal link with a quasi-natural experiment using a LCCPP in China, providing solid empirical evidence that links a direct positive relationship between environmental regulations and corporate green innovation. According to the findings, environmental regulation may promote innovation [71], supporting Porter’s "weak" argument.

Second, this research offers insightful information on how corporate green innovation is impacted by environmental regulation. This study introduces the moderating effects of external media concerns and organizational redundancy resources to understand the differential roles of internal and external governance contexts. This further enhances our understanding of the moderating effects of LCCPP on corporate green innovation. It also deepens the theoretical study of corporate and environmental governance.

Furthermore, while prior research has looked at how environmental regulation affects innovation, very few studies have concentrated on the mechanism behind this connection. Te effects of environmental regulation on innovation activities are caused by stability and financial restrictions. We find that LCCPP affects corporate green innovation by enhancing corporate green total factor productivity and financial stability, thus enriching the study of the mechanical effects of the benchmark relationship.

### 5.3 Managerial implications

This study has practical implications for promoting LCCPP and encouraging corporate green innovation.

A LCCPP can drive corporate innovation in green technologies, thereby promoting environmentally friendly and low-carbon development. Being a city-level environmental governance policy, this approach grants a certain degree of flexibility, enabling each pilot city to design its LCCPP plan based on local industrial structures and development priorities. The study findings indicate that the LCCPP plays a role in encouraging green technological innovation within enterprises. Therefore, policymakers in emerging economies can further encourage the implementation of LCCPP, and provide experience and reference for the carbon-neutral pilot projects proposed by the Ministry of Ecology and Environment. Given the weak constraints of the pilot policy, the government n emerging economies should supervise and guide the pilot cities during the implementation of the pilot policy, to induce enterprises to innovate in green innovation and achieve economic sustainability development.

Second, the information and supervision role of external media reports should be brought into play.The disclosure of environmental information operates as a digital interface, aligning businesses with diverse stakeholders, encompassing consumers and external investors. It plays a crucial role in fostering corporate innovation. Enterprises must acknowledge the significance of environmental information disclosure and proactively engage in disclosing such information to reduce information asymmetry and safeguard their corporate reputation. Moreover, companies should strive to enhance their environmental awareness and adopt a proactive approach to disclosing environmental information. This strategy can establish trust and support from stakeholders, leading to the efficient allocation of organizational resources and ultimately driving corporate green innovation.

Third, to achieve low-carbon cities, more explicit guidance programs for technological transformation should be developed for high-carbon industries to induce more innovative green invention-based innovations. The heterogeneity analysis in this study shows that implementing a pilot policy is more helpful in promoting green technological innovation in high-carbon industries than in low-carbon industries. The transformation of high-carbon industries is an important boost to the industrial restructuring of the pilot cities and the focus on achieving LCCPP construction. Only green innovation with higher value connotation is the key to promoting the transformation of high-carbon industries to low-carbon.

### 5.4 Limitations and future research

This paper introduces a novel theoretical framework for corporate green innovation, underscoring the key roles of both external media attention and internal organizational redundancy resources. Despite these contributions, it is important to acknowledge the presence of certain limitations within our research.

First, this study examines the pilot policy’s green innovation effects in LCCPP. Restricted by data availability, this study’s dynamic effect test shows a certain lag in the green technology innovation effect of the pilot policy, so a longer period of corporate green patent data should be tested. As the LCCPP continues to expand and gain momentum, it presents opportunities for future research to track and analyze the policy’s impact with the availability of more data. This enables researchers to conduct multidimensional studies and delve deeper into the subject.

Finally, it is important to note that this study has certain limitations regarding the mechanism for enhancing financial stability and the direct evidence of the impact of green finance policy on green technology innovation. Further exploration and testing are required in these areas. In terms of future research directions, potential moderating mechanisms such as political capital, environmental ethics or top management characteristics [72], which may affect the relationship between low-carbon city policies and corporate green innovation, warrant further investigation.

## Supporting information

S1 Data (XLSX)
